# Indication of diurnal variations in the rat choroid plexus for cerebrospinal fluid secretion

**DOI:** 10.1016/j.bbrep.2026.102674

**Published:** 2026-06-13

**Authors:** Takeshi Yamaguchi, Toshiyuki Hamada, Toshiyuki Matsuzaki, Norio Iijima

**Affiliations:** a Center for Medical Sciences, International University of Health and Welfare, 2600-1 Kitakanemaru, Ohtawara, Tochigi, 324-8501, Japan; b Department of Pharmacology, International University of Health and Welfare, 2600-1 Kitakanemaru, Ohtawara, Tochigi, 324-8501, Japan; c Department of Anatomy and Cell Biology, Gunma University Graduate School of Medicine, 3-39-22 Showa-machi, Maebashi, Gunma, 371-8511, Japan

**Keywords:** Choroid plexus, Cerebrospinal fluid, Diurnal variation, AQP1, CLDN2

## Abstract

The epithelial tissue of the choroid plexus (CP) is responsible for producing cerebrospinal fluid (CSF). The CP possesses a robust circadian clock, suggesting that CSF secretion from the CP is regulated by the circadian clock; however, the underlying mechanism is not yet fully understood. In the present study, we investigated diurnal variations in the expression of mRNA and proteins that contribute to CSF secretion in the rats. Of these gene products, only the expression of *Glut1* mRNA in the CP of the lateral and fourth ventricles (CP-LV and CP-4V, respectively) and *Cldn2* mRNA in the CP-4V exhibited significant diurnal variation using quantitative PCR analysis. On the other hand, the expression o*f Aqp1, Nkcc1, and Cldn2* mRNA showed fluctuation that alternate peaks and troughs every 3 h, and the timing of the peak in expression of the three genes was consistent. Western blot analysis did not reveal any significant diurnal variation in GLUT1 expression, whereas AQP1 in the CP-4V and CLDN2 in the CP-LV and CP-4V exhibited clear diurnal variation. The above results indicate that the diurnal variation observed in AQP1 and CLDN2 occur as the result of diurnal variations in translation or degradation activity. AQP1 and CLDN2 expression levels are likely to determine the changes in CSF secretion volume during a day. Fluorescence immunohistochemistry clearly detected AQP1 immunoreactivity in the apical side of the CP during the night, whereas almost no immunoreactivity was detected during the daytime. This result is suggestive of loss of antigenicity due to modified AQP1.

## Highlights

• *Aqp1*, *Cldn2*, and *NKCC1* mRNA showed short-term fluctuating expression in the rat choroid plexus (CP).
• CP AQP1 and CLDN2 protein expression was significantly higher in daytime.
• Regulation of translation/degradation may cause diurnal variation in these proteins.

## 1 Introduction

The circadian rhythm is a fundamental biological phenomenon that is common to most living organisms; it controls not only rest/activity rhythms but also various physiological functions, such as circulation and thermoregulation, via the autonomic nervous system in animals. In most animal cells, the circadian clock is driven by an interlocked transcription–translation feedback loop. The suprachiasmatic nucleus (SCN) in the mammalian hypothalamus is regarded as the most stable master clock in the body; circadian clocks outside the SCN have long been understood to be less stable than those in the SCN because of their one-sided, subordinate role in regulating circadian rhythms [1,2]. Recently, Myung et al. [3] reported that the expression of clock genes in the choroid plexus (CP) exhibits a consistent rhythm. Furthermore, Yamaguchi et al. [4] reported that clock gene exhibited diurnal variation only in epithelial cells of the CP. Myung et al. [3] also proposed that the circadian clock in the CP may reversibly regulate the clock in the SCN. Cerebrospinal fluid (CSF) is primarily produced by epithelial cells in the CP; its circulation in the ventricular system, spinal canal, and subarachnoid space is vital for maintaining homeostasis in the brain. Notably, CSF circulation is key to the removal of amyloid-beta, thereby contributing to the prevention of Alzheimer's disease. Moreover, CSF flow follows a diurnal pattern [[5], [6], [7]]. In the present study, we therefore further investigated the diurnal variations in gene product expression in the CP, focusing on proteins that are responsible for CSF secretion. The following proteins were examined: aquaporin-1 (AQP1), glucose transporter 1 (GLUT1), claudin-2 (CLDN2), ATPase Na^+^/K^+^ transporting subunit β1 (ATP1B1), Na^+^-K^+^-2Cl^−^ cotransporter 1 (NKCC1), transient receptor potential vanilloid 4 (TRPV4), and anoctamin-1 (ANO1) [8,9]. AQP1 is a member of the aquaporin family through which water moves passively along the osmotic gradient. AQP1 is primarily located on the apical membrane of CP epithelial cells of the lateral and fourth ventricles [[10], [11], [12], [13], [14]]. Although no diurnal rhythm was observed in *Aqp1* mRNA expression in the CP in our previous study [4], we examined both AQP1 protein and *Aqp1* mRNA more thoroughly in the present study. In addition, we investigated ATP1B1 [15]—a protein that, similar to AQP1, is primarily present on the apical membrane of CP epithelial cells and is responsible for CSF secretion—and ion channels such as NKCC1 [15,16], TRPV4, and ANO1 [9]. We also investigated GLUT1, which is primarily present on the basolateral side of the CP epithelium and drives osmotic gradient [17]. CLDN2 is an important component of epithelial tight junctions, which function as a blood–CSF fluid barrier. The epithelial tight junction formed by CLDN2 functions not only as a barrier but also as a paracellular pathway for small cations [[18], [19], [20]]. CLDN2 also facilitates the osmotic permeability of water [21,22].

In the current study, we examined diurnal variations in the expression of CSF-secreting gene products in the CP of the lateral ventricle (CP-LV) and the fourth ventricle (CP–4V) using the following three analyses: 1) day-long quantitative real-time polymerase chain reaction (qPCR), based on a report that the transcription of some CSF-secreting genes is directly regulated by clock gene proteins [8]; 2) day-long comparison of protein contents using Western blot analysis; and 3) immunohistochemistry against AQP1 to examine the distribution pattern of AQP1 protein.

## 2 Materials and methods

### 2.1 Animals

Male Wistar rats (8–10 weeks old) were purchased from Kumagai-shigeyasu Co.,Ltd. (Miyagi, Japan). The rats were housed at 21°C on a 12 h light/12 h dark cycle with *ad libitum* access to food and water. All experiments in the current study were conducted according to the National Institutes of Health Guidelines for the Care and Use of Laboratory Animals. The Committee of Animal Research of the International University of Health and Welfare approved the experiments (no. 17006).

### 2.2 Tissue dissection for qPCR and western blot

Four rats were sacrificed by decapitation every 3 h, at the following zeitgeber times (ZTs): 2, 5, 8, 11, 14, 17, 19, and 23. Anesthetic sevoflurane was administrated to the rats for sedation just before decapitation. After their brains were quickly removed, the CP-LV and CP-4V were extracted under a dissecting microscope.

### 2.3 Isolation of total RNA and quantitative real-time PCR

Each collected tissue (i.e., the CP-LV and CP-4V) was placed into 100 μL RNA Later (Thermo Fisher Scientific, Waltham, MA, USA) and stored immediately at 4°C. Total RNA was extracted from the CP-LV and CP-4V using TRIzol reagent (Invitrogen, Thermo Fisher Scientific, Waltham, MA, USA) and was then dissolved in 50 μL of RNase-free water and stored at −80°C. The cDNA was synthesized from total RNA of each sample using a ReverTra Ace qPCR RT Kit (Toyobo, Osaka, Japan). PCR was performed with TB Green Premix EX *Taq*II (TAKARA, Shiga, Japan) using a StepOnePlus system (Thermo Fisher Scientific); primer sequences are shown in Table 1. All reagents in this process were used according to the manufacturers’ instructions.

**Table 1:** Primer sequences used for qPCR.

Gene	Forward (5’ – 3′)	Reverse sequence (5’ – 3′)
*Aqp1*	CCCTCTTCGTCTTCATCAGC	CTGAGCCACCTAAGTCTCGG
*Glut1*	CTTCACTGTGGTGTCGCTGT	TTCAAAGAAGGCCACAAAGC
*Cldn2*	CGAGAAAGAACAGCTCCGTTT	TTCGCTTGTCTTTTGGCTGC
*Nkcc1*	AGATGACTTGCGGGAAGGTG	TGCCATCCTCTTCCTCATCTTT
*Trpv4*	GGGAACCATCCACAGGGAAG	CTGTCGCCTCATATCGGCTT
*Ano1*	GCAGGCCTGGAGCTGGAACG	GCTCAGCCACCTTGGGCTGG
*Atp1b1*	TGGAGACTTACCCTCTGACG	GGATTTCAGTGTCCAAGGTG
*Per2*	TTTGGGGGATGGGGTGAGATTC	TGATGAACTGAAGCCAGGTCCG
*Bmal1*	CCGATGACGAACTGAAACACCT	TGCAGTGTCCGAGGAAGATAGC
*Gapdh*	CTTCACCACCATGGAGAAGGC	GGCATGGACTGTGGTCATGAG

PCR was performed in duplicate, and RNA expression levels were calculated relative to the expression level of *Gapdh*, which was used as an internal control. Melting curve analysis of PCR products was performed after each amplification to demonstrate that only a single product was amplified. The PCR amplification profile was as follows: initial denaturation at 95°C for 30 s, followed by 40 cycles of denaturation at 95°C for 15 s, annealing and extension at 60°C (*Aqp1, Glut1*, *Cldn2, Nkcc1, Ano1, Atp1b1, Gapdh*) or 62°C (*Per2, Bmal1*) or 64°C (Trpv4) for 30 s. To determine whether the expression of each gene followed a diurnal variation, the peaks and troughs in the graph were statistically analyzed.

### 2.4 Western blot analysis

The CP-LV and CP-4V were homogenized before protein was extracted using an EzRIPA Lysis kit (ATTO Corporation, Tokyo, Japan) according to the manufacturer's protocol. The total amount of protein that can be obtained from the CP of a single rat is limited. Pre-measurement of protein concentration in each sample to equalize loading amounts was not performed, as this procedure could have resulted in an insufficient amount of protein remaining for Western blot analysis. Therefore, equal amounts of extracted protein were loaded into each lane, separated by 10% sodium dodecyl sulfate-polyacrylamide gel electrophoresis (SDS-PAGE), and subsequently transferred onto polyvinylidene fluoride (PVDF) membranes (Millipore, Burlington, MA, USA). After blocking with PVDF Blocking Reagent (TOYOBO, Osaka, Japan) at overnight at 4°C, the membranes containing transferred proteins were incubated with the following primary antibodies: AQP1 (1:2000, AffRaTM31, Matsuzaki laboratory, Gunma University, Maebashi, Japan) [23], CLDN2 (1:500, SAB4300737, MilliporeSigma, St Louis, MO, USA), GLUT1 (1:4000, Matsuzaki laboratory, Gunma University, Maebashi, Japan) [24], NKCC1 (1:10000, 13884-1-AP, Proteintech, Rosemont, IL, USA), and β-actin as an internal control (1:1000, bs-0061R, Bioss, Woburn, MA, USA) at overnight at 4°C. After washed with TBST for 3 times, the membranes were incubated with the following secondary antibody: rabbit immunoglobulin (Ig)G horseradish peroxidase–conjugated antibody (1:4000, #HAF008, R&D Systems, Minneapolis, MN, USA) for 1 h at room temperature. Protein signals were then visualized by chemiluminescence using Clarity Wester ECL Substrate (Bio-Rad, Hercules, CA, USA), and the chemiluminescent signals were captured using an ImageQuant LAS4000 Mini (GE Healthcare, Chicago, IL, USA). The expression levels of AQP1, CLDN2, GLUT1, and NKCC1 were normalized to the corresponding β-actin level in each individual sample, and subsequently quantified.

### 2.5 Statistics

Statistical analysis was performed using one-way analysis of variance (ANOVA) with a Bonferroni post-hoc test. Statistical significance was considered as P < 0.05.

### 2.6 Immunohistochemical analysis of AQP1 protein

At ZT2 and ZT17, rat brains were quickly removed and placed in ice-cold Hanks’ Balanced Salt Solution (MilliporeSigma). The CP-LV was removed from the brain using a dissecting stereomicroscope and preserved overnight in 4% paraformaldehyde in 0.1 M phosphate buffer (pH 7.0) The specimens were dehydrated through a graded ethanol series followed by xylene, and were then embedded in paraffin. Paraffin sections (2 μm thick) were prepared using a microtome (REM-7; Yamato Kohki Industrial Co. Ltd., Saitama, Japan) and firmly fixed onto glass slides (Platinum PRO; Matsunami Glass Ind. Ltd., Osaka, Japan). The sections were rehydrated through xylene and a descending series of ethanol. Next, sections were placed in 20 mM citric acid buffer (pH 6.0) and boiled for 20 min for antigen retrieval. After being treated with 5% normal goat serum in phosphate-buffered saline (PBS; Histofine SAB-PO kit; Nichirei, Tokyo, Japan) for 1 h, the sections were incubated for 4 days at 4°C with anti-AQP1 antibody (AffRaTM31, Matsuzaki laboratory, Gunma University, Maebashi, Japan) [23] diluted 1:1000 in PBS. The sections were then washed with PBS and incubated with goat anti-rabbit IgG H&L (Alexa Fluor® 488, Abcam, Cambridge, UK) for 1 h. The sections were washed with PBS and coverslipped using Fluoro-KEEPER Antifade Reagent with 4′,6-diamidino-2-phenylindole (Nacalai Tesque, Kyoto, Japan). A confocal laser scanning microscope (FV1200; Olympus, Tokyo, Japan) was used to observe the sections.

## 3 Results

### 3.1 Expression analysis of CSF secretion–related genes in the CP-LV and CP-4V using qPCR

**Fig. 1:**
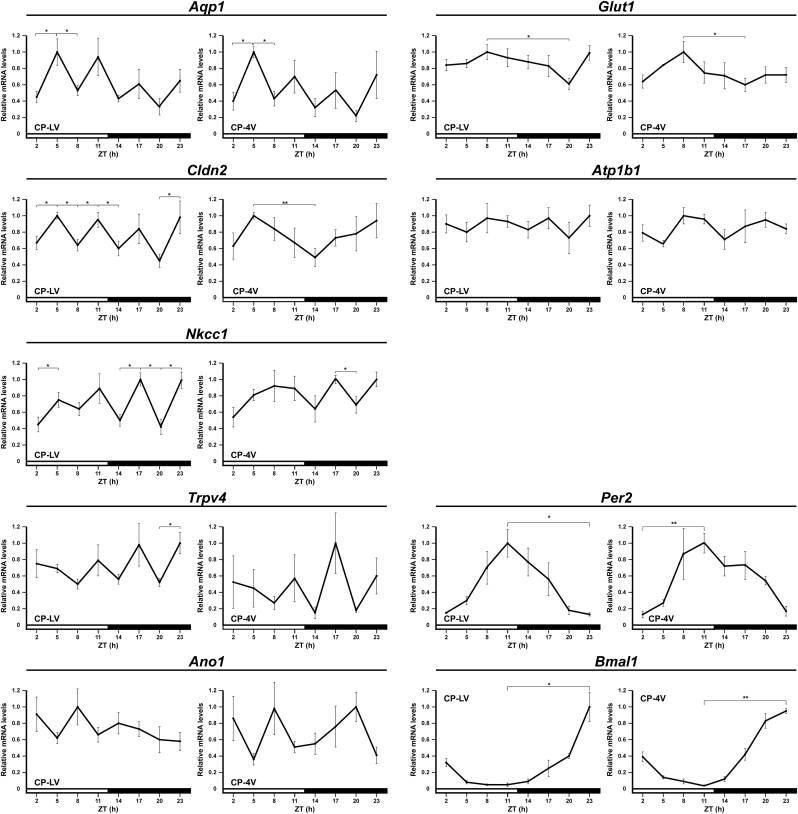
Daily expression patterns of genes relevant to CSF secretion (*Aqp1, Glut1, Cldn2, Nkcc1, Trpv4, Ano1,* and *Atp1b1*) and clock genes (*Per2* and *Bmal1*) in the CP-LV and CP-4V. Peak expression values of each mRNA are adjusted to 1.0 in each tissue. Values are expressed as the mean ± standard errors (*n* = 4). The expression of *Glut1* mRNA exhibited significant diurnal variation, whereas no other genes involved in CSF secretion were expressed with a meaningful diurnal cycle. Several mRNAs (*Aqp1, Cldn2,* and *Nkcc1*) were expressed in a fluctuating manner with a cycle of approximately 6 h, exhibiting peaks that coincided with each other. Statistical analysis was performed using one-way analysis of variance (ANOVA) with a Bonferroni post-hoc test (*P < 0.05 and **P < 0.01, respectively).

The expression patterns of *Glut1* mRNA in the CP-LV and CP-4V and *Cldn2* mRNA in the CP-4V showed alternating peaks and troughs, with one peak during the day and one trough at night; the peak values were significantly higher than the trough values (Fig. 1). By contrast, the expression patterns of other mRNAs showed no clear differences in peak and trough values throughout the day and night. Interestingly, however, the expression pattern of *Aqp1* mRNA in the CP-LV and CP-4V alternated between peaks and troughs at 3 h intervals. Similar expression patterns were observed for *Cldn2* mRNA and *Nkcc1* mRNA in the CP-LV. Within a single day, significant differences were observed between some adjacent peaks and troughs in the expression of each mRNA. Additionally, the peak expression patterns of each mRNA over 6 h intervals overlapped with each other. As a control experiment, the mRNA expression of *Per2* and *Bmal1* was examined using the same RNA samples. The mRNA expression of *Per2* and *Bmal1* followed a diurnal rhythm in both the CP-LV and CP-V4; *Per2* peaked once during the day and *Bmal1* peaked once at night. These findings indicate that the tissue sampling and RNA extraction processes were performed appropriately in the current study.

### 3.2 Expression analysis of CSF secretion–related proteins in the CP-LV and CP-4V

**Fig. 2:**
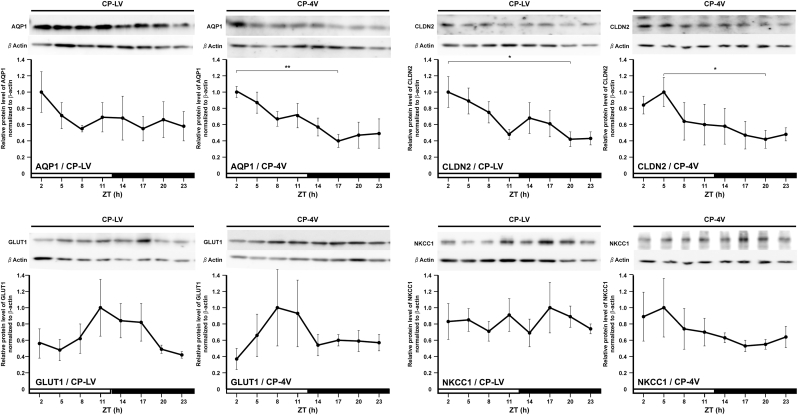
Daily expression of AQP1, CLDN2, GLUT1, and NKCC1 protein in CP-LV and CP-4V. The expression level of each protein was normalized to the corresponding β-actin level in each sample. Peak expression values of each protein are adjusted to 1.0 in each tissue. Values are expressed as the mean ± standard error (*n* = 4). AQP1 protein was expressed in the CP-4V with significant diurnal variation. CLDN2 protein was expressed in the CP-LV and CP-4V with significant diurnal variation. Statistical analysis was performed using one-way analysis of variance (ANOVA) with a Bonferroni post-hoc test (*P < 0.05 and **P < 0.01, respectively).

The highest expression levels of AQP1 protein in the CP-4V and CLDN2 protein in the CP-LV and CP-4V during the day were significantly higher than the respective lowest expression levels at night (Fig. 2). A large variance in expression levels was observed in AQP1 protein in the CP-LV and in GLUT1 and NKCC1 proteins in the CP-LV and CP-4V, with no significant differences in expression levels between the day and night.

### 3.3 Immunohistochemical analysis of AQP1 protein in the CP-4V

**Fig. 3:**
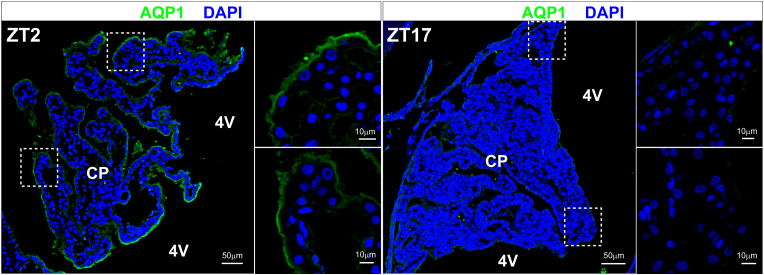
Immunofluorescent staining against AQP1 in the CP-4V at ZT2 (left) and ZT17 (right). AQP1 was detected on the apical side of epithelial cells in the CP-4V at ZT2.

Immunoreactivity for AQP1 was most prominent in the apical side of the CP-4V at ZT2 (Fig. 3, left), whereas, almost no signal was observed at ZT17(Fig. 3, right).

## 4 Discussion

The flow of CSF contributes to the clearance of amyloid-beta peptide deposits in the brain, thus helping to prevent Alzheimer's disease. It follows a diurnal rhythm in both mice and humans [5,7]; flow is faster during the rest phase than during the active phase. The rats which are nocturnal animals were used in this study whose CSF is secreted most actively during the day when they are resting. Several hypotheses have been proposed regarding the source of CSF secretion and the driving force behind its flow [6,[25], [26], [27]]. Given that the CP is the oldest known CSF-secreting capillary network, we hypothesized that the CP secretes CSF in a diurnal rhythm, and CSF therefore circulates with diurnal variations. We planned this study based on the hypothesis that increased expression of proteins involved in water channels that cause water movement, proteins involved in solute movement, and the genes for these proteins enhances CSF secretion.

*Glut1* mRNA expression exhibited significant diurnal variation in the CP-LV and CP-4V. However, large individual differences in protein expression levels hindered the determination of a diurnal rhythm from these fluctuations in expression levels. The limited sample size did not provide clear indication of diurnal variation. No significant day-night changes or regularity difference in *Trpv4*, *Ano1*, and *Atp1b1* mRNA expression were observed. Western blotting was omitted in this study because reliable antibodies against the three gene products were not available.

This study revealed that the levels of AQP1 and CLDN2 proteins, which were known to be directly involved in water osmotic permeability, exhibited diurnal fluctuations. Similar to the diurnal variation observed in *Cldn2* mRNA in the CL-4V, CLDN2 protein expression exhibited a diurnal rhythm in the CP-4V. In this case, the amount of mRNA is presumed to reflect the amount of protein directly. On the other hand, the expression patterns of *Aqp1* mRNA in the CP-LV and CP-4V, and of *Cldn2* mRNA in the CP-LV, exhibited a fluctuating appearance with alternating peaks and troughs every 3 h. This pattern of expression was observed for three genes, including *Nkcc1* mRNA, with overlapping peak timing, suggesting that these transcriptional mechanisms may share a common regulatory function. Further studies are expected to show that the expression of these mRNAs in the CP follows a strict 6 h sinusoidal curve, which could be called an ultradian rhythm. It remains unclear whether this short-term fluctuation in transcription is directly linked to the robust circadian clock of CP. However, further studies will help shed light on the mechanism behind this particular expression pattern. In line with these findings, a 4 h ultradian rhythm in gene expression has previously been reported in the vicinity of the hypothalamus [28]. These results suggest that there are two major transcriptional mechanisms for the CSF secretion-related factors investigated in this study: one that causes short-term fluctuations in transcription levels, and the other that causes diurnal variations. The functioning transcription machinery varies depending on the tissue, and it varies depending on gene even in the same tissue.

The expression levels of AQP1 protein in the CP-4V and CLDN2 protein in the CP-LV differed significantly between the day and night; however, no significant differences were observed in the mRNA expression levels. This is presumably because of differences in the translational activity of the genes or in the half-lives of the proteins between the day and night. Although it remains unclear whether these changes in the expression of AQP1 and CLDN2 proteins are directly dependent on the robust circadian clock present in the CP, the diurnal fluctuations in these proteins are highly likely link directly to diurnal fluctuations in CSF secretion in the CP. Regarding the relationship between the levels of these proteins and CSF production, it has been reported that CSF production is reduced in AQP1-deficient mice, suggesting that the expression level of AQP1 protein is directly linked to the regulation of CSF secretion [29].

In our previous immunohistochemical study [4], we reported that no difference was observed in AQP1 expression levels in the CP between day and night in experiments using strongly enhanced DAB staining, therefore in this study we used a fluorescent antibody staining method, which was more quantitative properties than DAB staining. At ZT2, AQP1 protein was strongly expressed in the apical side of the CP, as in previous studies, whereas its expression was barely detectable at ZT17. This result is inconsistent with the Western blot results showing that AQP1 expression at ZT17 was half that at ZT2. Extremely decreased immunoreactivity at ZT17 may be due to changes in antigenicity caused by modifications of the AQP1 protein. Such modification may also be reflected in the decrease in daytime CSF secretion via the water channel activity of AQP1.

## 5 Conclusion

In the present study, the levels of AQP1 protein in the CP-4V and CLDN2 protein in the CP-LV and CP-4V were significantly higher during the daytime resting period than during the nighttime active period. This difference in protein levels between day and night may hypothetically be responsible for the increased daytime secretion of CSF. Interestingly, the expression patterns of some CSF-secreting genes appeared to follow short-term expression fluctuations. Furthermore, immunofluorescence staining for AQP1 revealed that the immunoreactivity of AQP1 disappeared at night, suggesting that the antigenicity of AQP1 was altered by modification.

## Funding

This work was financially supported by the Japan Society for the Promotion of Science (JSPS) KAKENHI [grant number 24K12098 to TY and 24K03322 to NI].

## CRediT authorship contribution statement

**Takeshi Yamaguchi:** Data curation, Formal analysis, Funding acquisition, Investigation, Visualization, Writing – original draft. **Toshiyuki Hamada:** Writing – review & editing. **Toshiyuki Matsuzaki:** Resources, Writing – review & editing. **Norio Iijima:** Conceptualization, Funding acquisition, Investigation, Project administration, Supervision, Writing – original draft.

## Declaration of competing interest

The authors declare the following financial interests/personal relationships which may be considered as potential competing interests: Norio Iijima reports financial support was provided by Japan Society for the Promotion of Science. Takeshi Yamaguchi reports financial support was provided by Japan Society for the Promotion of Science. If there are other authors, they declare that they have no known competing financial interests or personal relationships that could have appeared to influence the work reported in this paper.

## Data Availability

No data was used for the research described in the article.
