# Supplementary material for: Indication of diurnal variations in the rat choroid plexus for cerebrospinal fluid secretion
Source: Biochem Biophys Rep. 2026 Jun 13;47:102674. doi: 10.1016/j.bbrep.2026.102674 (PMC13276760; doi:10.1016/j.bbrep.2026.102674)
Supplement: Multimedia component 1 [file mmc1.pdf]

AQP1 / CP-LV

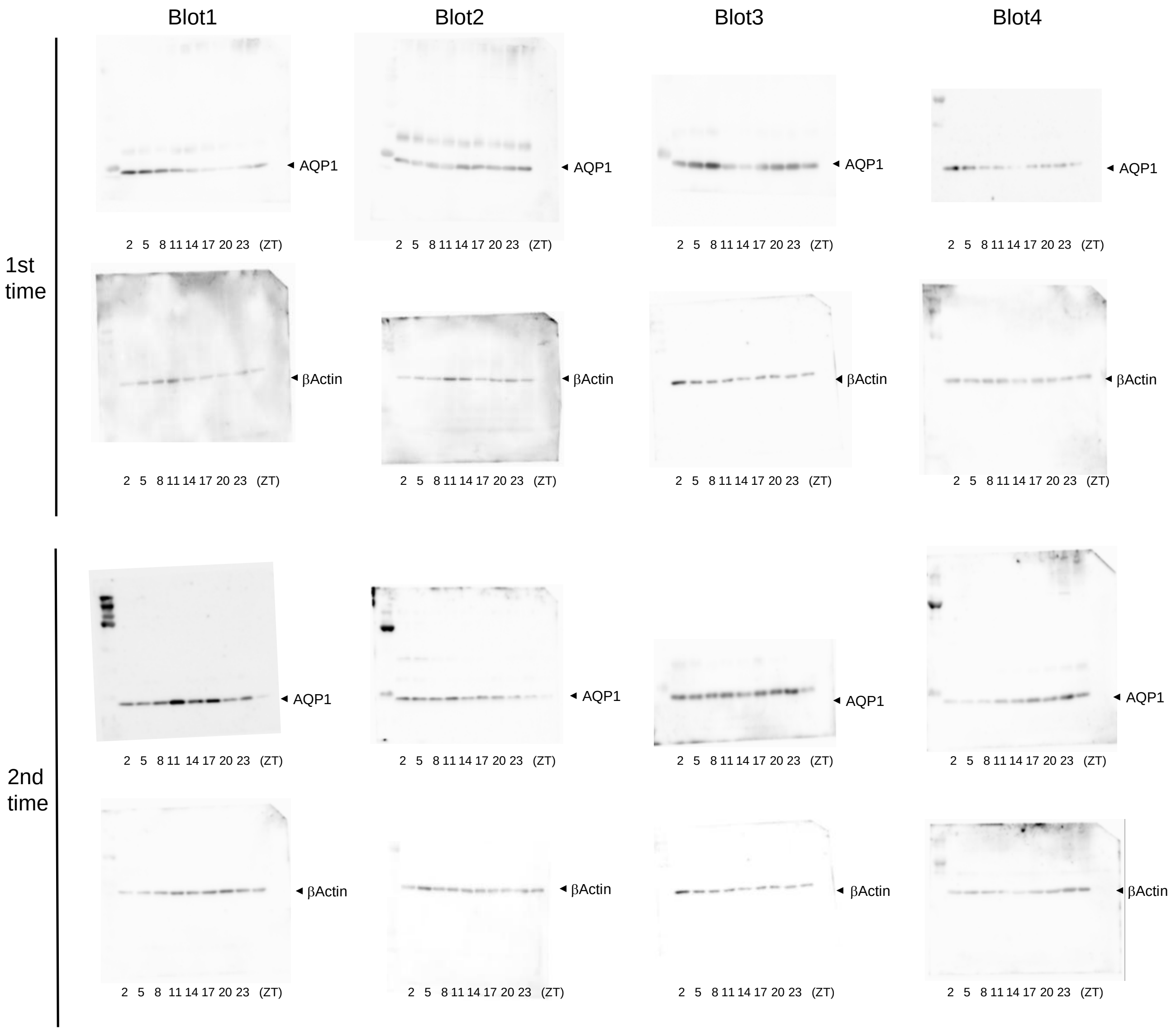

Supplementary figure 1.

All original Western blot images used for the detection of AQP1 (top) and internal control β-actin (bottom) in CP-LV, which were used for the quantitative analysis shown in Figure 2.

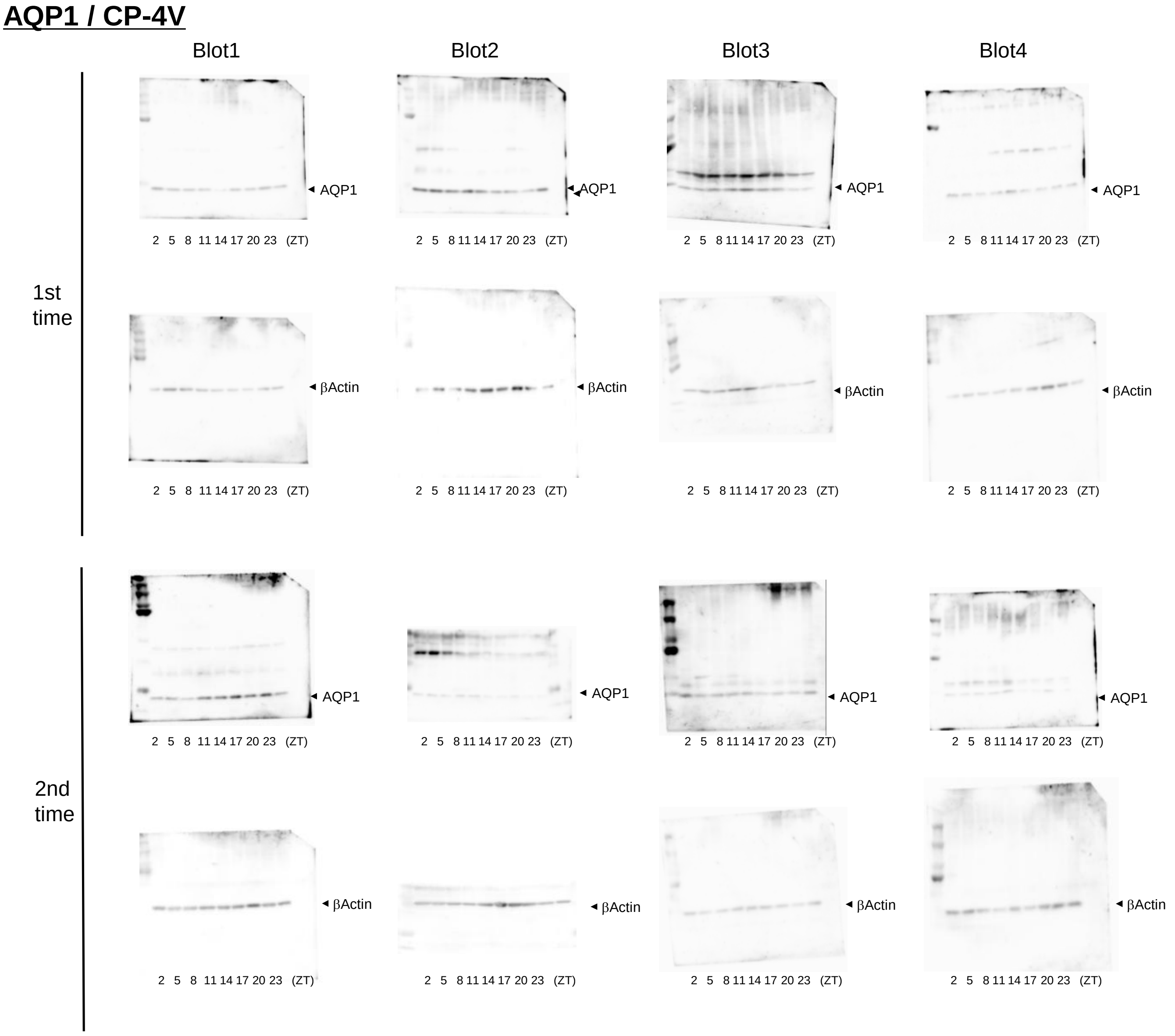

Supplementary figure 2.

All original Western blot images used for the detection of AQP1 (top) and internal control β-actin (bottom) in CP-4V, which were used for the quantitative analysis shown in Figure 2.

CLDN2 / CP-LV

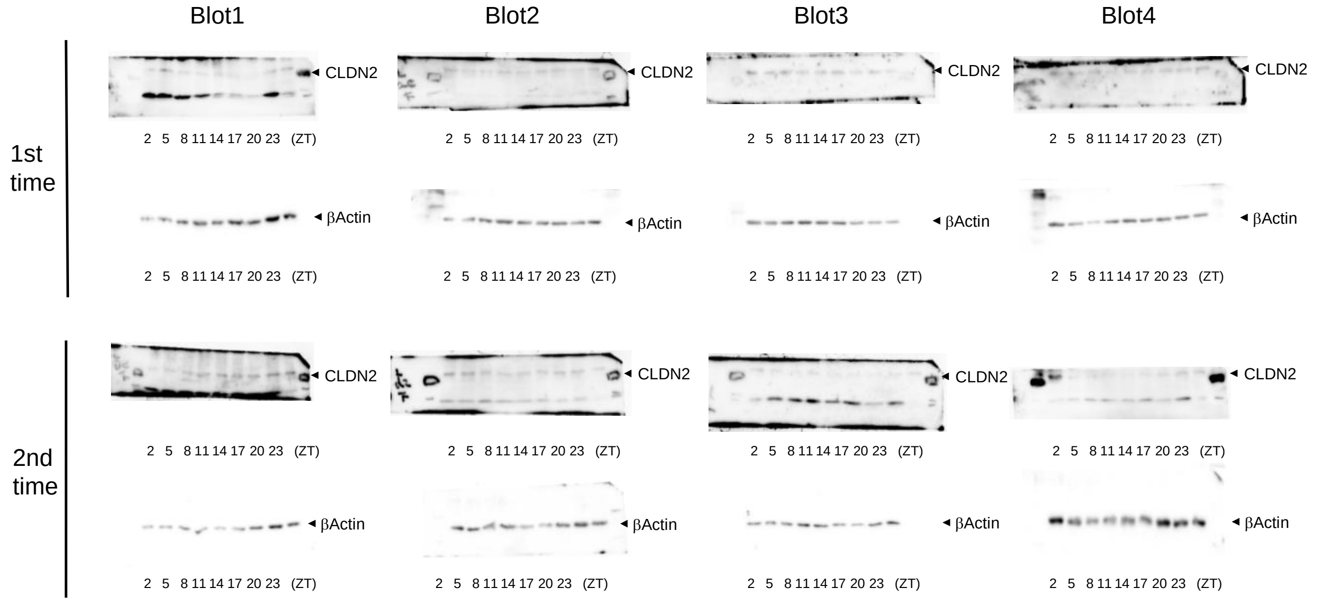

Supplementary figure 3.

All original Western blot images used for the detection of CLDN2 (top) and internal control β-actin (bottom) in CP-LV, which were used for the quantitative analysis shown in Figure 2.

CLDN2 / CP-4V

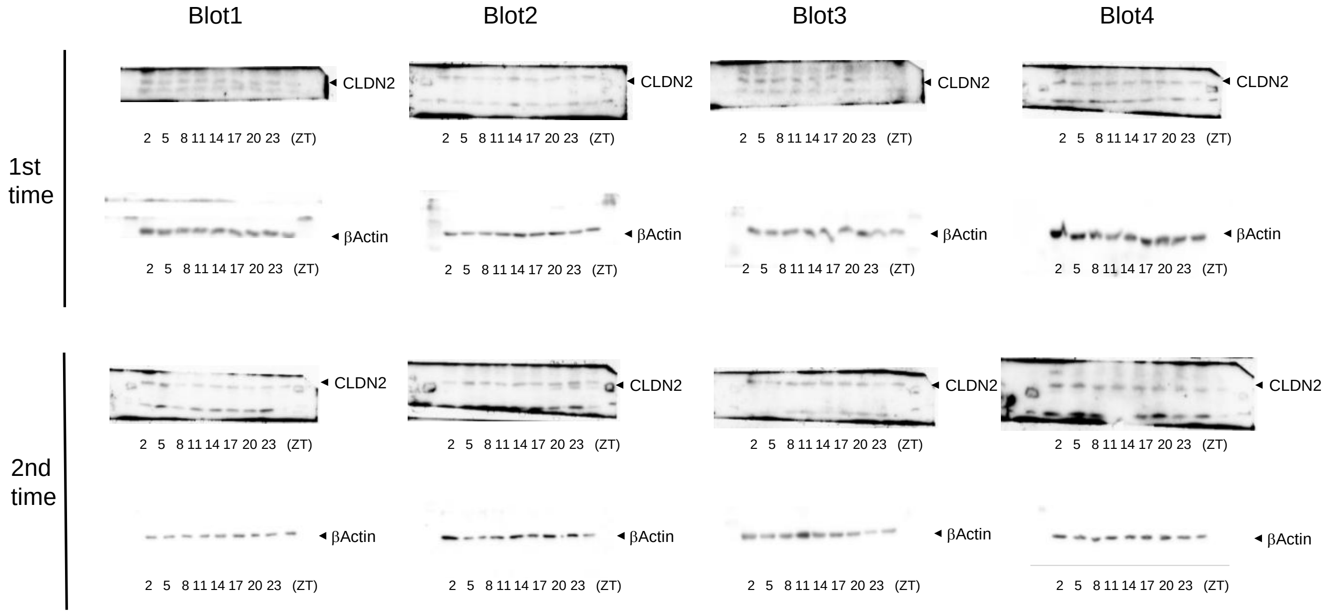

Supplementary figure 4.

All original Western blot images used for the detection of CLDN2 (top) and internal control β-actin (bottom) in CP-4V, which were used for the quantitative analysis shown in Figure 2.

GLUT1 / CP-LV

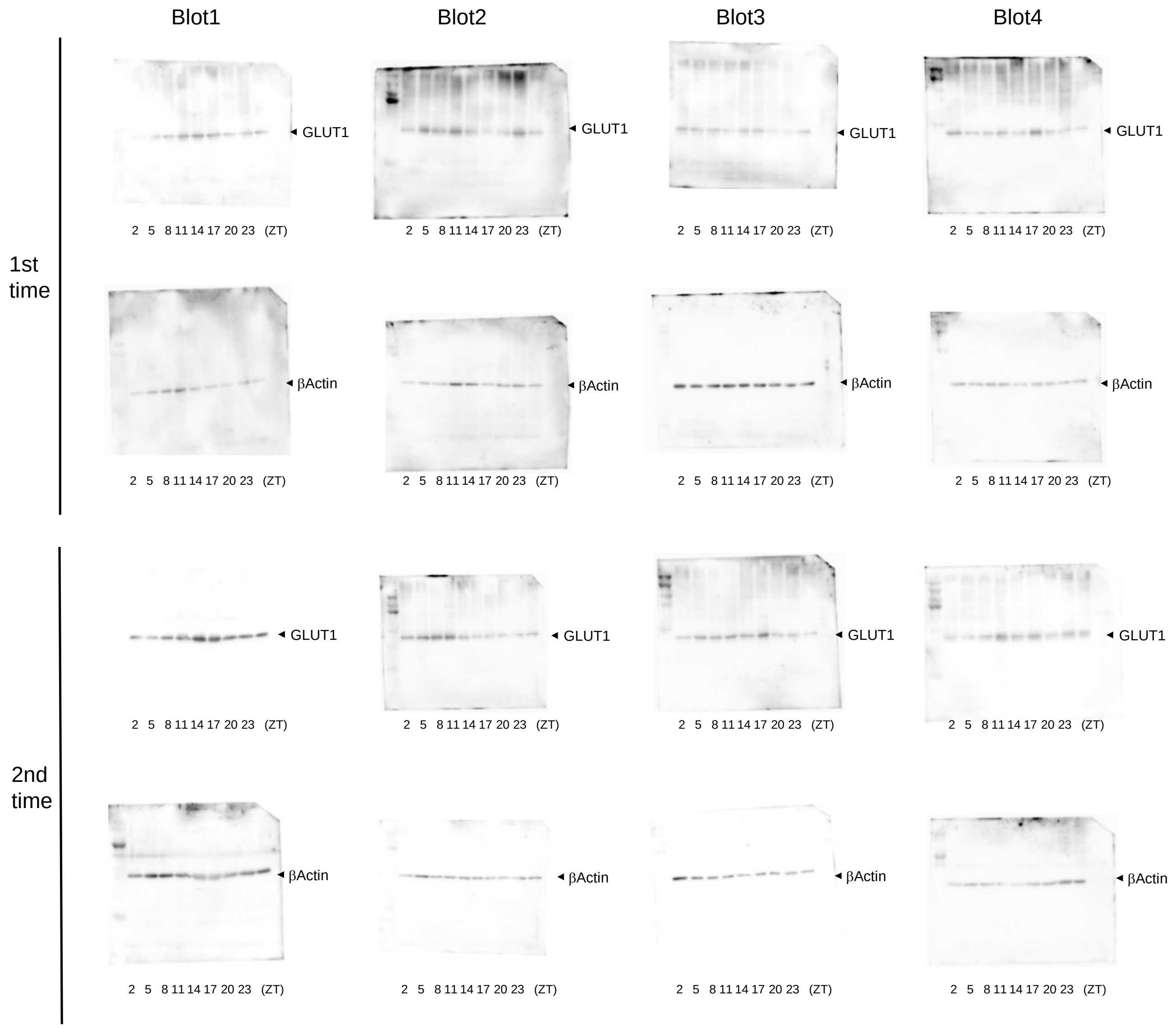

Supplementary figure 5.

All original Western blot images used for the detection of GLUT1 (top) and internal control β-actin (bottom) in CP-LV, which were used for the quantitative analysis shown in Figure 2.

GLUT1 / CP-4V

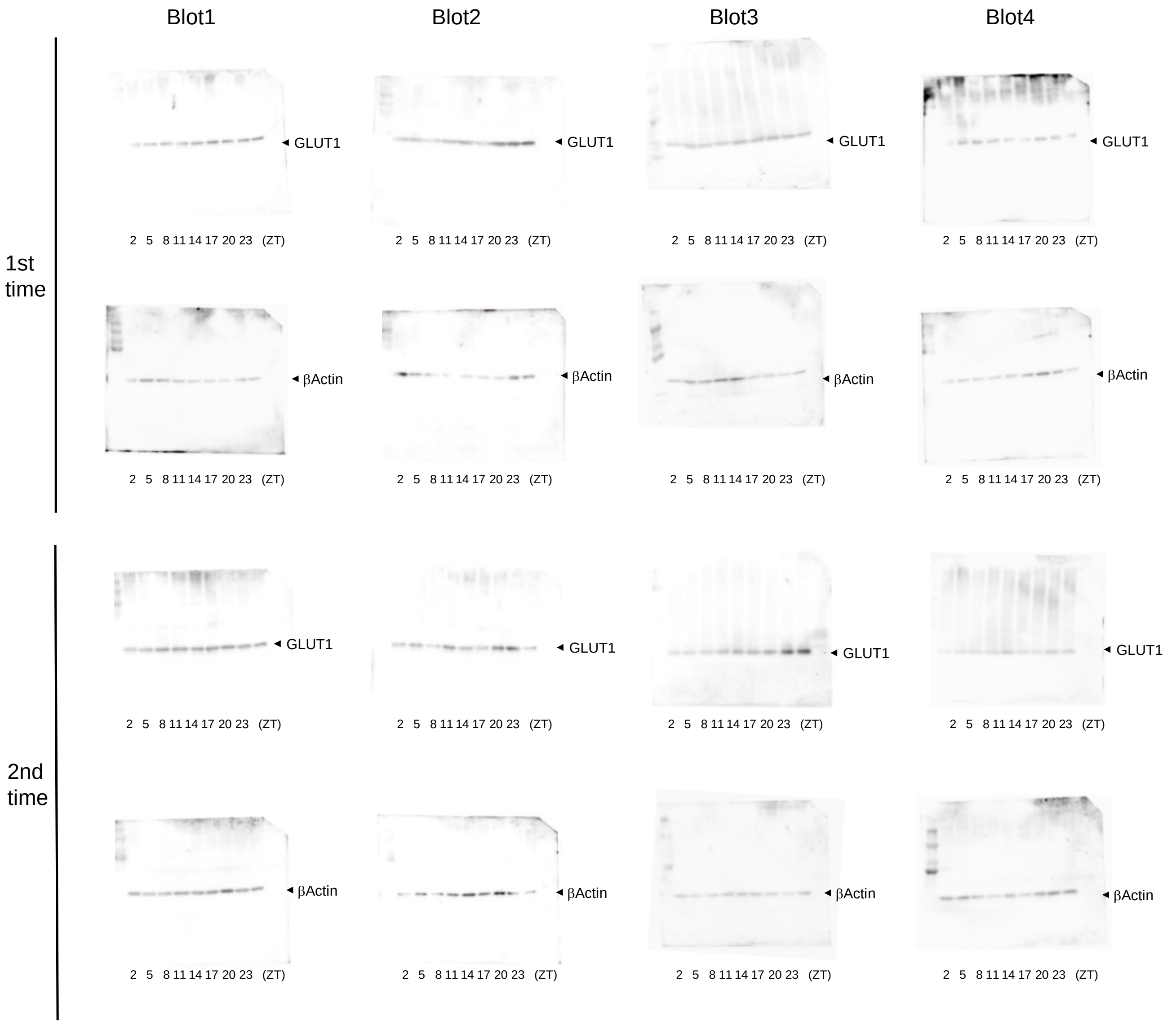

Supplementary figure 6.

All original Western blot images used for the detection of GLUT1 (top) and internal control β-actin (bottom) in CP-4V, which were used for the quantitative analysis shown in Figure 2.

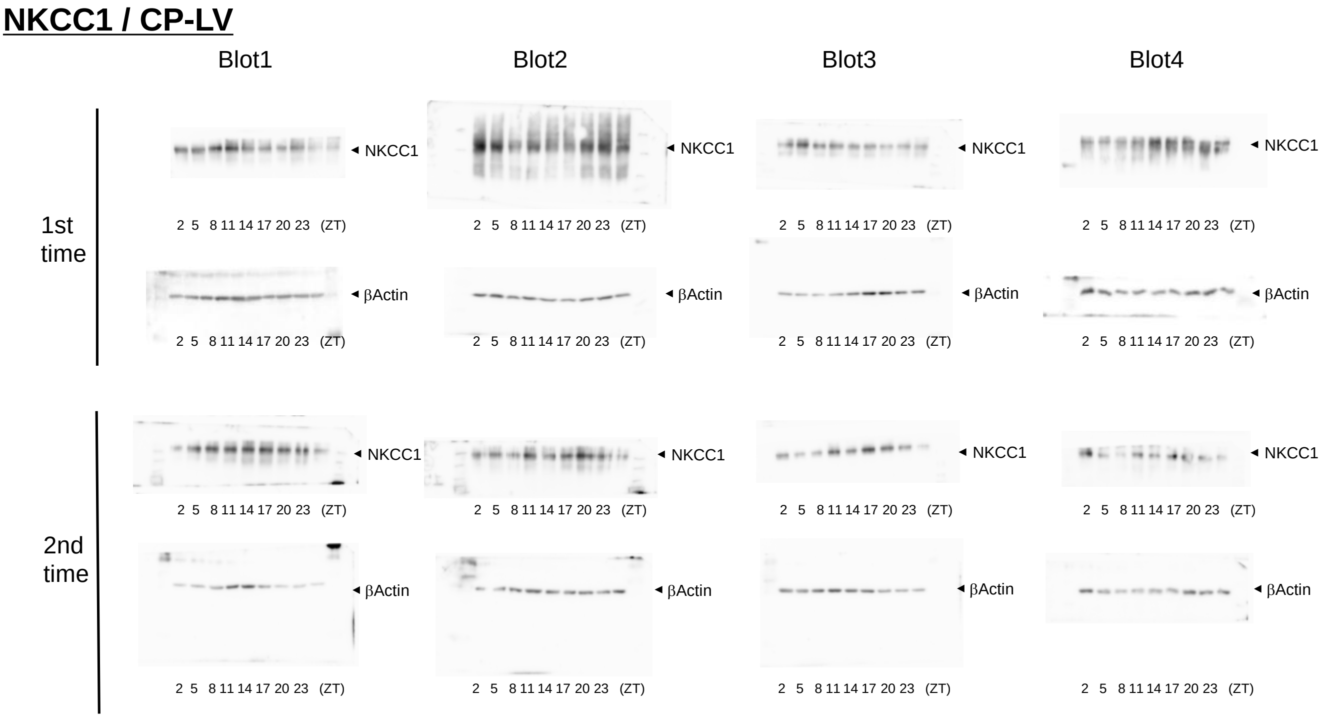

Supplementary figure 7.

All original Western blot images used for the detection of NKCC1 (top) and internal control β-actin (bottom) in CP-LV, which were used for the quantitative analysis shown in Figure 2.

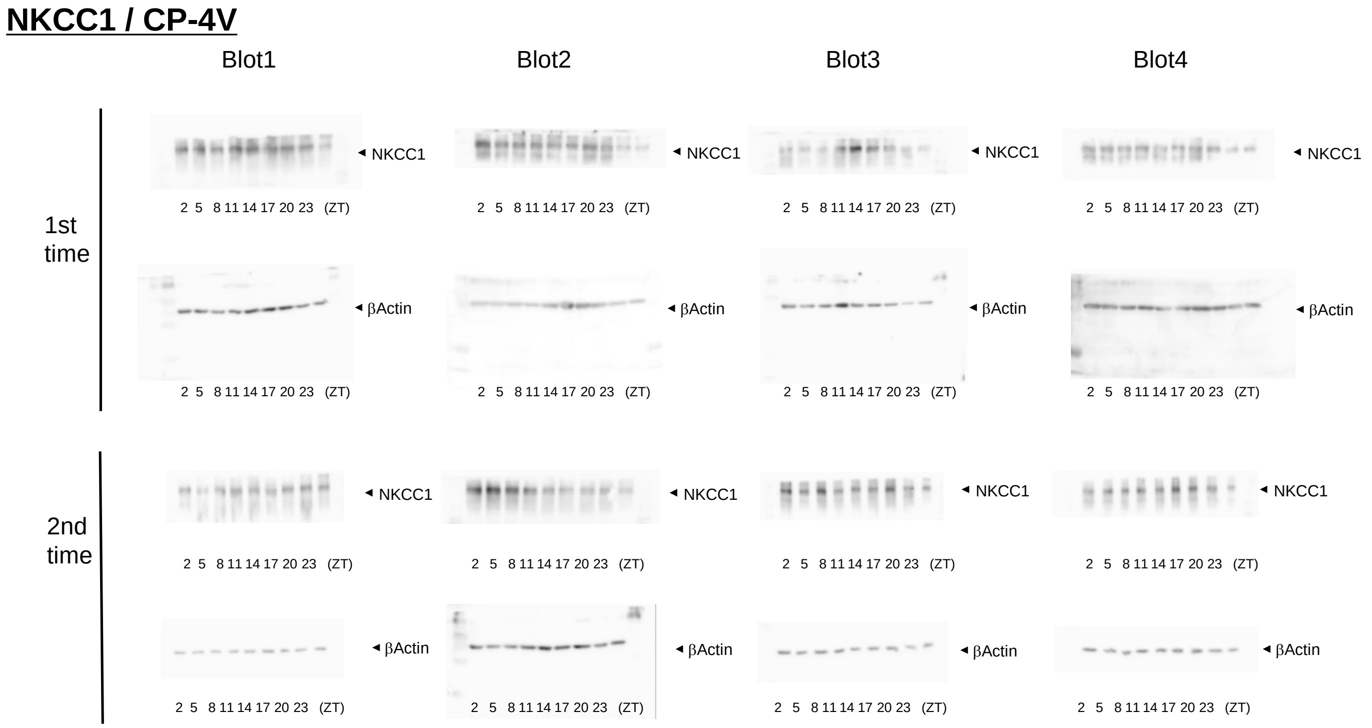

Supplementary figure 8.

All original Western blot images used for the detection of NKCC1 (top) and internal control β-actin (bottom) in CP-4V, which were used for the quantitative analysis shown in Figure 2.
